# Desiccation induces viable but Non-Culturable cells in *Sinorhizobium meliloti *1021

**DOI:** 10.1186/2191-0855-2-6

**Published:** 2012-01-20

**Authors:** Jan AC Vriezen, Frans J de Bruijn, Klaus R Nüsslein

**Affiliations:** 1 Plant Research Laboratory-DOE, Michigan State University, East Lansing, Michigan, USA, MI 48824; 2 Department of Microbiology, University of Massachusetts, Amherst, Massachusetts, USA, MA 01003; 3 CNRS-INRA, Laboratoire des Interaction Plantes Micro-organismes (LIPM), Castanet Tolosan, CEDEX, France

**Keywords:** *Sinorhizobium meliloti*, Desiccation, Live/Dead, Viable But Non-Culturable

## Abstract

*Sinorhizobium meliloti *is a microorganism commercially used in the production of *e.g*. *Medicago sativa *seed inocula. Many inocula are powder-based and production includes a drying step. Although *S. meliloti *survives drying well, the quality of the inocula is reduced during this process. In this study we determined survival during desiccation of the commercial strains 102F84 and 102F85 as well as the model strain USDA1021.

The survival of *S. meliloti *1021 was estimated during nine weeks at 22% relative humidity. We found that after an initial rapid decline of colony forming units, the decline slowed to a steady 10-fold reduction in colony forming units every 22 days. In spite of the reduction in colony forming units, the fraction of the population identified as viable (42-54%) based on the Baclight live/dead stain did not change significantly over time. This change in the ability of viable cells to form colonies shows (i) an underestimation of the survival of rhizobial cells using plating methods, and that (ii) in a part of the population desiccation induces a Viable But Non Culturable (VBNC)-like state, which has not been reported before. Resuscitation attempts did not lead to a higher recovery of colony forming units indicating the VBNC state is stable under the conditions tested. This observation has important consequences for the use of rhizobia. Finding methods to resuscitate this fraction may increase the quality of powder-based seed inocula.

## Introduction

Rhizobia form root nodules in symbiosis with legumes in which they fix atmospheric nitrogen and supply the fixed nitrogen to the plants (Jones et al. 2007). This system can be used to replenish soils with biologically-fixed nitrogen, and reduces the need for chemical fertilizers and pollution. However, this process is affected by salinity, drought and desiccation stress (Zahran 1999; Vriezen et al. 2007). To make optimal use of this system, inoculants are employed which allow for close contact between the microorganisms and the germinating seed (Smith 1992; Kosanke et al. 1999; Deaker et al. 2004). Many inoculants are powder-based, and a drying step during production reduces their quality (Kosanke et al. 1992). According to Catroux et al. (2001) many inoculants remain unreliable because of the inability of bacterial cells to persist under adverse conditions, including desiccation.

Many conditions have been identified that affect the survival of agriculturally important *Rhizobiaceae *during desiccation (Vincent et al. 1962; Bushby and Marshall 1977 Some factors affecting the survival of root nodule bacteria on desiccation; van Rensburg and Strijdom 1980; Dye 1982; Salema et al 1982 Death of rhizobia on inoculated seed, 1982 Rupture of nodule bacteria on drying and rehydration; Kremer and Peterson 1983; Mary et al. 1985, 1986; Kosanke et al. 1992; Smith 1992; Chenu 1993; Boumahdi et al. 1999; Estrella et al. 2004; Kaci et al. 2005; Vriezen et al. 2006). These conditions include the intrageneric differences to cope with desiccation stress affecting survival (vanRensburg and Strijdom 1980; Mary et al. 1985, 1986; Trotman and Weaver 1995; Boumahdi et al. 1999; Sadowski and Graham 1998). All of these studies estimated the surviving fraction of cells using plate count techniques, however, it is possible that the number of surviving cells is not affected but just the ability to form colonies. For a long time it has been known that cells can enter a Viable But Non-Culturable (VBNC) state, which includes rhizobia (Manahan and Steck 1997, Alexander et al. 1999, Basaglia et al. 2007). The VBNC state is a state in which cells are viable, but do not form colonies. The induction of this physiological state is very relevant to field practices since rhizobial VBNC cells do not infect plants, while those able to form colonies do (Basaglia et al. 2007).

To be able to understand the fate of microorganisms in seed inocula that require a drying step, rhizobial cells need to be investigated under the limiting conditions they experience under desiccation stress. Therefore, we studied the fate of *S. meliloti *cells during desiccation stress from three days to nine weeks. We not only determined the number of surviving cells that are able to form colonies on regular growth media, but also estimated the surviving cells using direct counting techniques including the live/dead stain. Based on differences in results between these enumeration techniques, the results indicate that only a fraction of the living rhizobial cells are able to form colonies after desiccation suggesting that desiccation induces a VBNC state. The finding that desiccation induces a VBNC state in rhizobia has not been reported in the literature before. Finding methods to resuscitate the VBNC population may greatly enhance culturability of rhizobia, thus the quality of powder-based seed inocula.

## Materials and methods

### Materials, strains, growth and drying media

*S. meliloti *1021 (Meade et al. 1982) was taken from our culture collection. Commercially available strains of 102F84 and 102F85 from the Novozyme collection (strain NRG-43 and NRG-185 by Rice (1982) respectively) were provided by R.S. Smith. The media used were Tryptone Yeast extract (TY, Beringer 1974), Yeast Extract Mannitol Broth (YMB, Atlas and Parks 1993), and Phosphate Mannitol Medium (PMM, Vriezen et al. 2006). The matrices used were Ottawa Sand Standard (1 g/microcentrifuge tube, 20-30 mesh, Fisher Scientific, Fairlawn, New Jersey), alfalfa seeds were previously sterilized (Vriezen et al. 2006) and guaranteed untreated. (1 g/microcentrifuge tube, Outsidepride, BS-ALFALFA-5; Lot No: A2N-1769-3) and 0.45 μM nitrocellulose filters (25 mm diameter; Millipore, HA02500, Bedford, MA).

### Assay to measure survival during desiccation

Survival during desiccation was determined using a method developed previously (Vriezen et al., 2006). Briefly, TY medium was inoculated and incubated with bacteria for three days at 28°C, while agitated at 220 rpm until full cell density was reached (OD_595 nm _~ 2.5). Fifty microliters of this culture were transferred to tubes containing each five milliliters of YMB or PMM, and incubated with agitation at 28°C. Resulting exponentially growing cultures were incubated until an OD_595 nm _= 0.1-0.4 was reached, and concentrated by centrifugation. The pellet was resuspended in one milliliter of sterile YMB or PMM medium and pipetted 100 μL onto a scaffold, which here is a nitrocellulose filter, sand or seed. Nitrocellulose filters are historically used for drying rhizobia in studies addressing the responses of said organism to desiccation (Mary et al. 1985 and 1986, Boumahdi et al. 1999). In previous studies, we found a relation of cell number with desiccation survival. When using nitrocellulose filters, this is not seen. The three different matrices were placed into a microcentrifuge tube and stored at a relative humidity (RH) of 22% for three days at 20°C in the dark. 22% RH was chosen because survival of *S. meliloti *RCR2011, the parent of *S. meliloti *1021, is highest between 67%-22% RH (Marie et al, 1985). Thus for studying physiological responses, drying at 22% RH represents severe desiccation conditions without detrimental effects of drying too fast or too severe. 22% RH in the air phase was obtained by drying in airtight glass jars over a saturated solution of potassium acetate (Vriezen et al, 2006).

To determine the number of surviving cells, the filters were removed from the jars and exposed to 100% RH for one hour inside an enclosed container at room temperature since exposure to water saturated air increases the colony forming units (Data not shown). One milliliter YMB medium was added to the sample while inside the microcentrifuge tube, and the CFU were determined by plate counting on YMB.

### Microscopic examination

To study the possibility of irreversible binding to the filter, the recovery efficiency of cells from the filters was determined using plate counts and phase contrast microscopy. A volume of 50 μL of non-diluted crystal violet (Difco 4312525) was added to one milliliter of bacterial suspension to enhance the contrast. Also the live/dead staining kit (Baclight, Invitrogen, Carlsbad, CA) was used to estimate relative numbers of dead and living recovered cells making use of the selective permeability of cell membranes for propidium iodide (Anonymous 2004; Basaglia et al. 2007). This assay is based on the differential ability of two nucleic acid dyes to penetrate the cell. The green fluorescent dye penetrates all bacterial cells, while the larger red fluorescent dye binds the DNA of only those cells it can penetrate, i.e. those with compromised membranes. Resuspended cells were mixed 1:1 in 1 mL solution containing 3 μL Syto9 and 3 μL propidium iodide. 25 μL of this solution were transferred onto a microscopic slide coated with LE Agarose (Genepure, ISC Bioexpress, Kaysville, Utah). LE agarose was used to immobilize cells to aid with direct counting (Liu et al. 2001). After 15 minutes incubation in the dark, the numbers of live and dead cells were counted microscopically using epifluorescent illumination (Olympus BX51, Olympus USA, Center Valley, PA) with the appropriate filter blocks. At least three frames per sample were completely counted with a minimum of one hundred cells.

### Mathematical methods

The percentage of surviving cells are displayed as the ^10^Log of this percentage, where 2 = 100% survival and 0 = 1% survival. Regression lines were calculated and line graphs made using Excel (Microsoft Office Excel 2003 SP2). The F-test was used to determine differences in variance, the T-test to determine levels of significance using Excel. SEM = Standard Error of the Mean. For further calculations, please see Additional file 1.

## Results

### Survival during desiccation of three *S. meliloti *strains

**Figure 1:**
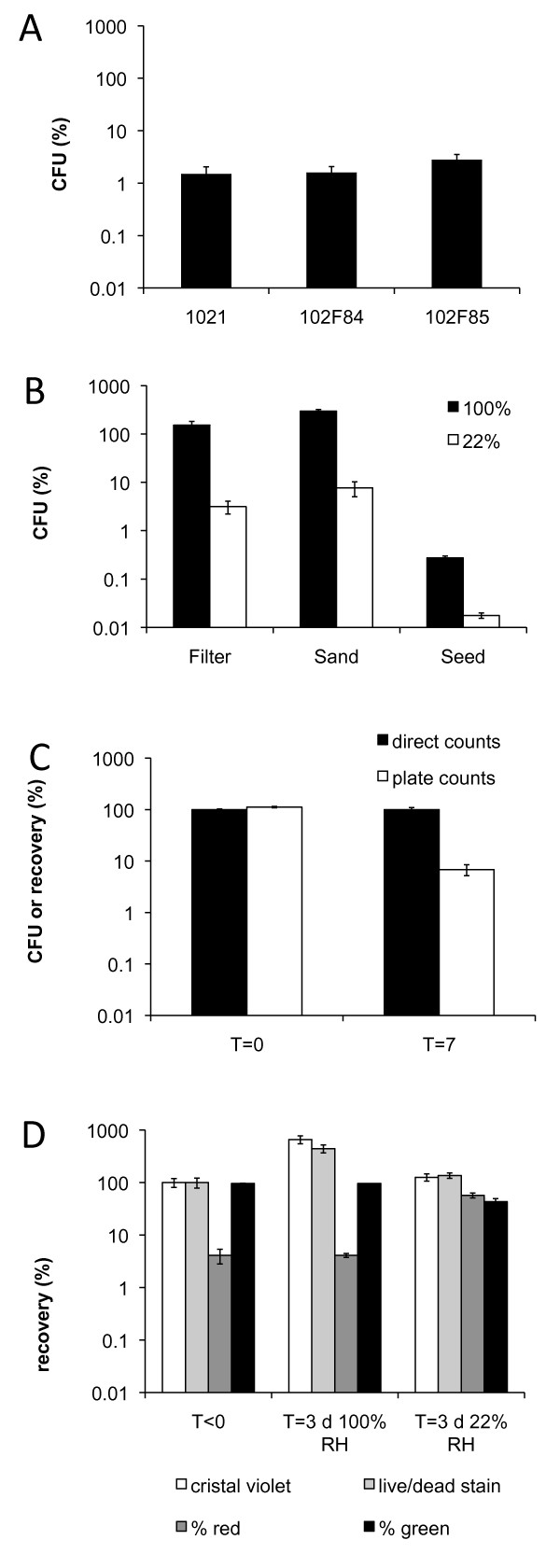
**Survival and recovery of *S. meliloti *cells after drying and rewetting**. (A) Survival (CFU) of *S. meliloti *strains 1021, 102F84, and 102F85 in PMM, dried on nitrocellulose filters and stored at 22% relative humidity (RH) for three days (n = 45, 18, 18, respectively). (B) Survival (CFU) of *S. meliloti *1021 on nitrocellulose filters, Ottawa sand, or alfalfa seeds. Cells were stored at either 100% (black bar) or 22% RH (white bar) for five days (n = 6). (C) Survival (CFU, white bar) and cell recovery (direct counts, black bar) of cells using a nitrocellulose filter over seven days (n = 6). (D) Quantitative recovery of cells (direct count) after three days of storage under 100% or 22% RH on nitrocellulose filters (white bar = cristal violet, light grey bar = Live/dead, dark grey bar = %red, black bar = %green). In all graphs the error bars represent the SEM.

Two commercially available *S. meliloti *strains were tested for their ability to survive desiccation and compared to the survival of model organism *S. meliloti *1021 (Figure 1A). Survival of strain 1021 at 1.51% is not significantly different than that of strain 102F84 with 1.61% (P > 0.10). 2.80% of cells of strain 102F85, survived, which is 1.9 and 1.7 fold better than strain 1021 and 102F84, respectively (P < 0.10).

### Identification of a suitable matrix for desiccation studies

To discriminate between matrix and desiccation effect, three matrices were tested for their effect on survival of *S. meliloti *1021 during desiccation.": (i) nitrocellulose filters, (ii) Ottawa Sand, and (iii) alfalfa seeds. Controlled storage conditions without drying were achieved using the same matrices, but the matrices were kept in air above water leading to 100% RH in the air-phase. The colony forming units (CFU) of surviving cells on a nitrocellulose filter in Ottawa Sand and on alfalfa seeds was negatively affected during storage at 22% RH (Figure 1B). When dried in Ottawa Sand and on nitrocellulose filters, the CFU declined to 7.7% and 3.1% of the original CFU respectively (Figure 1B), and did not differ significantly (P > 0.05). When *S. meliloti *cells were dried on alfalfa seeds, the reduction in colony forming units was much greater, and only 0.018% survived (a 5.7 × 10^3 ^fold reduction, Figure 1B). This reduction includes both a matrix as well as an adverse effect caused by desiccation. At 100% RH, the number of CFU was not affected negatively in sand nor on filters, however, when placed on sterile alfalfa seeds, CFU did decline to 0.27%. This can be explained by a toxic effect of the seed-surface, which has been described previously (Salema et al. 1982 Death of rhizobia on inoculated seed, Smith 1992, Vriezen et al. 2006, Penna et al. 2011). We chose to further explore the responses of *S. meliloti *1021 to desiccation using nitrocellulose filters, since the error on filter is lower compared to the use of a sand matrix, and no negative matrix effect is seen similar to drying on alfalfa seeds. Most importantly, since the focus of this investigation was the physiological response to desiccation of *S. meliloti*, the ideal matrix does not affect survival but only desiccation does

### Microscopic examination using differential staining

Desiccation over seven days affected the culturability of the cells (Figure 1C). After seven days at 22% RH the number of CFU dropped to 6.8% compared to the direct count. In comparison, after the same time but at 100% RH there was no significant loss in the numbers of CFU or total cells.

Since only a fraction of the cells form colonies after desiccation, we expected that those cells that lost the ability to form colonies have a compromised cell membrane. To determine the role of desiccation in the loss of cell membrane integrity, the BacLight Live/dead stain was employed on the cells recovered from nitrocellulose filters. Using this stain one can discriminate between cells that lost membrane integrity, stain red and are dead, while intact viable cells stain green (See Additional file 2). At 100% RH, the number of dead cells (4.4 ± 0.5%) had not changed significantly after three days compared to experimental onset (Figure 1D). However, after drying over three days, the number of dead cells (red) increased to 56.9 ± 10.6%, while the remaining 43.1% of the cells stained green. During that time there was no significant difference between direct microscopic counts supported by the contrast enhancing dye crystal violet and direct counts with the BacLight stain.

### Long term storage

**Figure 2:**
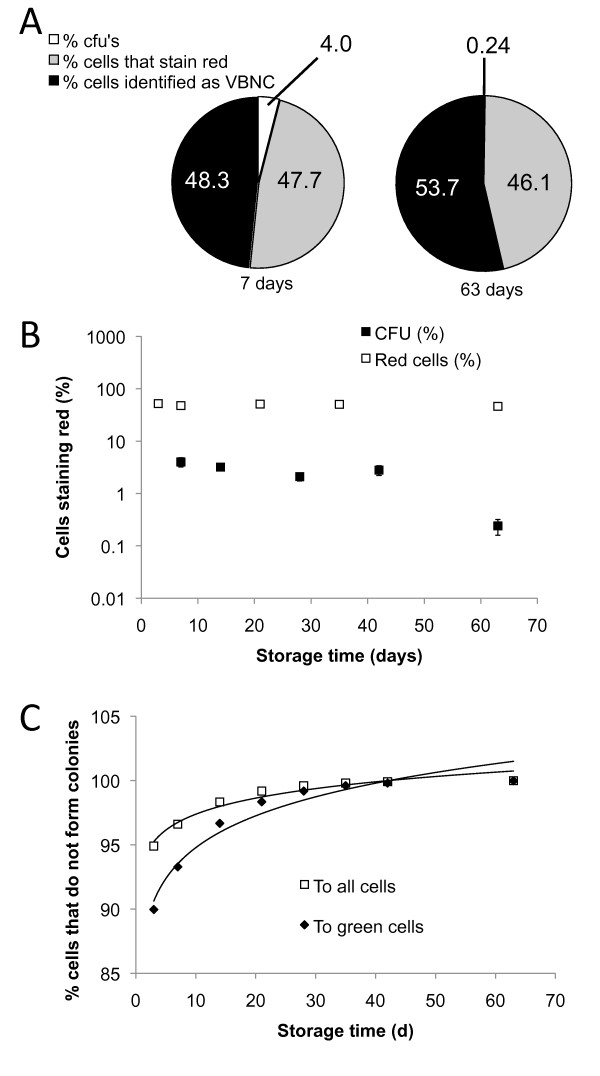
**Fate of the recovered *S.meliloti *1021 cells after desiccation**. (A) Comparison of the fate of recovered cells after desiccation and stored for seven or 63 days (white sector = %CFU, grey sector = %cells that stain red, black sector = %cells identified as VBNC). The difference between the membrane-compromised fractions at both time points is not significant (P > 0.05). (B) Comparison of the fate of recovered *S. meliloti *1021 cells over nine weeks of storage. Markers represent: black square = %CFU (Samples taken at T = 7, 14, 28, 42, 63 days with n = 27, 105, 102, 74, 27 respectively) and the white square = %dead (Red) cells (n = 10 per time-point). Error bars represent SEM. (C) Fraction of cells that lost the ability to form colonies over nine weeks. The number of VBNC cells were calculated relative to total cell number and the number of cells that stained viable. The deduced fraction of VBNC relative to green cells is represented by a black diamond and relative to all cells is white square.

After long-term desiccation, we found that the fraction of cells that are able to form a colony decreased from 4.0% (SEM = 0.8) after seven days to 0.24% (SEM = 0.08) after 63 days (Figure 2A). Direct microscopic observation using BacLight staining showed that, after seven days, an estimated 47.7% of cells stained red, thus, are dead (SEM = 2.3) and 48.3% of the cells are viable but do not form colonies. After 63 days, the fraction of viable cells was 53.7%, while 46.1% stain red and thus are dead (SEM = 2.3). There was no significant difference in the number of dead (red) cells between these time points (P > 0.05). We annotated the fraction of viable cells that lost their ability to form a colony the Viable But Non-Culturable fraction, or VBNC. In this experiment, after seven days, the VBNC fraction relative to the total number of green cells is 100-(4.0/52.3 × 100) = 92.4%. After 63 days, this VBNC fraction is 99.6% (Figure 2A). This fraction may contain VBNC cells that can be resuscitated (Maraha, 2007).

To estimate the rate of loss of culturability over time, changes in the CFU as well as in the dead fraction of cells during a time course of 63 days was determined (Figure 2B). The CFU declined as a function of time following the formula: Y(%CFU) = 6.9 × 10^-0.044X ^(R^2^= 0.74). The fraction of dead cells followed the regression line: -0.0605X+50.985 (R^2 ^= 0.37). Thus, when integrating different time points the CFU declined steadily, while the fraction of viable cells remained similar over time. A regression line describing the deduced increase of the VBNC fraction, relative to the green fraction of cells, was determined as 86.6 × X^0.039 ^(R^2 ^= 0.95, Figure 2C). Relative to the total number of cells the VBNC increased by 93.3 × X^0.018 ^(R^2 ^= 0.95, Figure 2C).

### Estimation of storage time

The initial decrease in surviving cells and the rate of decline of CFU during prolonged desiccation both can be combined to estimate the time it takes until all cells have lost the ability to form colonies (Table 1). The survival times using nitrocellulose filters and alfalfa seeds were estimated to reach a final CFU of < 1 (complete loss of culturability) or the final CFU of 1000 (required colony number for seed inocula by French and Canadian standard, Smith 1992) starting with 1.0 × 10^7 ^cells (the estimated starting CFU at T = 0 in our experiments). We also calculated the loss based on the initial CFU of 2.0 × 10^9^, based on the estimated number of CFU at T = 0 by Boumahdi et al. (1999). We concluded, that complete loss of culturability is achieved after 128 ± 2 days of desiccation storage. The estimated lower limit of cells required in inocula for alfalfa seeds will be reached in 62 ± 1 days.

**Table 1:** Estimated storage time in days during desiccation on two matrices to achieve target CFU of 1000 or 1

	Matrix*					
	Nitrocellulose		Ottawa Sand		Alfalfa seed	
Initial reduction (IR^†^)	41.6 ± 7.5		33.0 ± 12.8		(6.2 ± 2.2) × 10^3^	
Target CFU (N_f_^‡^)	1	1000	1	1000	1	1000
Initial CFU (N_0_^§^)						
1 × 10^7^	128 ± 2	60 ± 2	134 ± 5	66 ± 5	78 ± 1	10 ± 1
2 × 10^9^	181 ± 2	113 ± 2	187 ± 5	118 ± 5	131 ± 1	62 ± 1

(*) Error represent the Standard Error of the Mean with n = 6

(†) IR = Initial Reduction (N_t0-5 _= N_0_/N_t _= 5)

(‡) N_f _= Target CFU. Complete loss (< 1 CFU), French Canadian Standard = 1000 CFU (Smith 1992)

(§) N_0 _= Initial CFU

## Discussion

The symbiotic system of rhizobia with legumes can be used to replenish soils with biologically-fixed nitrogen and prevents the need for chemical fertilizers and pollution. To make optimal use of this system, inoculants are employed allowing close contact between the microorganisms and the germinating seed (Kosanke et al. 1999; Smith 1992; Deaker et al. 2004). Powder-based seed inocula require a drying step to enable long-term storage. This drying step can result in a reduction in inoculum quality (Smith 1992), i.e. reduced culturability and infectivity. Although culturability is reduced, a substantial part of the cells are in a viable but non-culturable state (VBNC). The induction of this physiological state by desiccation is a novel and very relevant observation since rhizobial VBNC cells do not infect plants, while those able to form colonies do as reported for strain *S. meliloti *41 (Basaglia et al. 2007).

This physiological state, in which bacterial cells are alive, but not able to form colonies, exists in many microorganisms, including rhizobia (Manahan and Steck 1997; Alexander et al. 1999; Basaglia et al. 2007). Such a state was first described by Xu et al. (1982), and has been extensively reviewed (Oliver 2005; Maraha 2007; Hayes and Low 2009; Barcina and Arana 2009). In this study we used the Baclight live/dead stain to discriminate between living cells and dead cells. While it seems accurate to assume that membrane compromised bacterial cells can be considered dead (Nebe-von-Caron et al. 2000; Anonymous 2004; Berney et al. 2006, 2007), the reverse, that intact cells are active cells is not necessarily true (Joux and Lebaron 2000; Berney et al. 2007). Although a small fraction of cells with compromised membranes will be able to repair their membranes and grow, and some cells scored as intact will not be able to grow (Berney et al. 2007; Giao et al. 2009), this differential cell stain remains the method of choice for VBNC studies. The term VBNC applies directly to the observations presented in this manuscript. Cells of *Sinorhizobium meliloti *1021, that were rewetted following desiccation, can be divided into three groups: (i) viable cells that form colonies, (ii) viable cells that do not form colonies, the VBNC fraction, and (iii) dead cells which lost membrane integrity.

Group I and II: Many environmental factors have been identified inducing a VBNC state in bacteria, which are natural stress, temperature stress, osmotic upshift, oxygen stress, white light, pasteurization, and chlorination (Oliver 2005). Also desiccation can induce a VBNC state in *E. cloacae *(Pederson and Jacobsen 1993) and *S. meliloti *(this manuscript). The VBNC fraction contains two groups of cells, the temporarily non-culturable and the permanently non-culturable (Maraha 2007). The challenge is to find the conditions that lead to resuscitation of the temporarily non-culturable fraction. Barry et al. (1956) noted that autoclaving media leads to an increase in H_2_O_2 _preventing growth. Therefore the addition of sodium pyruvate and catalase to the medium increased resuscitation in many organisms (Mizunoe et al. 1999; Imazaki and Nakaho 2009). We also attempted to resuscitate cells by plating and counting on different media (TY and YMB), with the addition of catalase or sodium pyruvate, and investigated different plating methods, regular and plate drop methods (data not shown). We were not able to increase the number of CFU amongst the cells that we considered to be in the VBNC state. Basaglia et al. (2007) shows that lack of O_2 _leads to a VBNC state in *S. meliloti *strain 41, another model organism, however, O_2 _supplementation alone does not result in resuscitation, nor did the addition of catalase, which is in agreement with our results. We also tested the effect of oxic versus anoxic conditions on survival but found that it did not increase CFU over 63 days of storage at 22% RH (data not shown). Based on these findings it appears that all VBNC cells are in a permanent state of non-culturability. Manahan and Steck (1997) showed that tap water induced the VBNC state in *S. meliloti *1021, however, the inducing component may have been copper in the tap water as was later shown by Alexander et al. (1999) for *A. tumefaciens *and *R. leguminosarum*.

Group III: Desiccation leads to an increase in the fraction of cells that lost membrane integrity. This desiccation induced loss of membrane integrity can be caused by changes in phase transition of phospholipids (Potts 1994; Leslie et al. 1995), by hypo-osmotic stresses during rewetting and the consequent breakage of the cell wall (Bushby and Marshall 1977 Desiccation induced damage to the cell envelope of root nodule bacteria, 1977 Some factors affecting the survival of root nodule bacteria on desiccation; Salema et al. 1982 Rupture of nodule bacteria on drying and rehydration), and by lipid per-oxidation when cells are not able to repair damages during low water availability (Borst et al. 2000; Billi and Potts 2002). Currently, it is not known which of the above listed processes are the underlying mechanisms for our observations.

Another possible explanation for the occurrence of VBNC cells after desiccation is that these cells are without functional template for replication of DNA. DNA is a major target of desiccation in microorganisms, including *E.coli *(Asada et al. 1979) and *D. radiodurans *(Mattimore and Battista 1996), as degradation of DNA is common as a response to desiccation. In rhizobia, desiccation sensitive mutants were isolated with mutations in the DNA repair systems (Humann et al. 2009), confirming that rhizobia are similarly affected by desiccation. It would be interesting to see if the CFU of rhizobia increases after desiccation with *in-trans *expression of DNA repair enzymes.

In this study we estimated the effect of storage time during nine weeks of desiccation of *S. meliloti *1021. The reduction of CFU is 0.044 ^10^Log per day, a 10-fold decrease per 22 days. Using our data, it is possible to estimate the storage time to reach sterility or the required lower limit for successful seed inoculation of 1000 CFU/seed. *E.g*. when dried on nitrocellulose, a sample containing 10^7 ^CFU would lead to sterility in 128 days (Table 1). However, when 2 × 10^9 ^bacteria per filter are assumed (for easy comparison the same amount of *S. meliloti *RCR2011 cells was taken which Boumahdi et al. (1999) had placed on a filter), sterility or the minimum of 1000 CFU (Smith 1992) would be reached after 113 days, and the complete loss of CFU after 181 days. When dried on the seed, the time it takes *S. meliloti *1021 to reach the standard of 1000 CFU is 10 days, and 62 days with an initial cell number of 10^7 ^and 2 × 10^9^, respectively. Since this is substantially shorter than the estimates by Boumahdi et al. (1999), the estimated storage time can not be estimated by only using survival rates after the initial decline in CFU, but should take into account the fast decrease in CFU during the early phases of drying. Additionally, when dried in the lag- and stationary phase on a filter under 22% RH of the air phase Boumahdi et al. (1999) found *S. meliloti *2011 to survive for 457 and 138 days, respectively. We determined a storage time of 113 days. This study extended the excellent findings by Boumahdi et al. (1999). The discrepancies between the results can be explained by the differences in the methods. In contrast to our method, Boumahdi et al. (1999) used filter suction and a much higher concentration of cells. Unfortunately, this group did not provide a bi-phasic survival curve, or used lag-phase cells in their storage time estimations. Furthermore, it is unclear if the cells they had used were growing cells or diluted late stationary phase cells. This is important, since the physiological differences between the two types are expected to have different responses to desiccation (Vriezen et al. 2006).

The observation and study of the appearance of VBNC cells is extremely important for the interpretation and risk assessment of data regarding the fate of rhizobial cells in soil and seed inocula. Our study shows that (i) desiccation induces a VBNC state in *S. meliloti*, which has not been reported before; (ii) finding methods to avoid a VBNC state may improve the quality of dry base seed inocula, considering the finding by Basaglia et al. (2007) that only resuscitated VBNC cells can successfully infect plant roots; (iii) the commercial *Sinorhizobium meliloti *strain 102F85 survives short term desiccation better than strains 102F84 and 1021, and (iv) underestimation of survival may have consequences for the spread of introduced rhizobia.

## Competing interests

The authors declare that they have no competing interests.

## Supplementary Material

Additional file 1 **Mathematical methods**. This file contains an explanation of the mathematical methods used throughout the manuscript. Click here for file

Additional file 2 **Example of bac/light direct counting results**. Photographic image of Syto9 and Propidium iodine stained cells stored at 100% RH (A) and after drying and rewetting (B). Click here for file
