# Supplementary material for: Desiccation induces viable but Non-Culturable cells in Sinorhizobium meliloti 1021
Source: AMB Express. 2012 Jan 20;2:6. doi: 10.1186/2191-0855-2-6 (PMC3293009; doi:10.1186/2191-0855-2-6)
Supplement: Additional file 1 — Mathematical methods. This file contains an explanation of the mathematical methods used throughout the manuscript. [file 2191-0855-2-6-S1.PDF]

Desiccation induces Viable But Non-Culturable Cells in *Sinorhizobium meliloti* 1021

Applied and Industrial Microbiology Express

Jan A. C. Vriezen[1,2], Frans J. de Bruijn[1,3] and K. Nüsslein[2*]
[1]*Plant Research Laboratory-DOE, Michigan State University, East Lansing, Michigan, USA, MI 48824*
[2]*Department of Microbiology, University of Massachusetts, Amherst, Massachusetts, USA, MA 01003*
[3]*CNRS-INRA, Laboratoire des Interaction Plantes Micro-organismes (LIPM), Castanet Tolosan, CEDEX, France*

ADDITIONAL FILES

Additional file 1: Mathematical methods.

<u>The parameters defined</u>:

T = Time (days)
N_0 = Initial number of bacterial cells (CFU)
N_5 = number of bacterial cells at T=5 days (CFU)
N_f = final number of bacterial cells (CFU)
T_f = Time (days) when N_f is reached
T_5 = Time (days) when N_5 is reached
% survival = $(N_f/N_0)\mathrm{x}100$
Y = % CFU
Y_5 = % CFU_{T5}
Y_f = % CFU_{Tf}
N_r = number of red cells (dead cells)
N_g = number of green cells (living cells)
N_t = total number of cells = N_r+N_g

<u>Initial reduction rate over the first five days:</u>

Initial Reduction at T_5 = $IR=N_0/N_5 \leftrightarrow N_5=N_0/IR$
% survival at T_5 = $(N_5/N_0)\mathrm{x}100$
% survival at T_f = $(N_f/N_0)\mathrm{x}100$

<u>Long term rates from day five until day 63</u>:

Long term survival (%CFU) regression line: $Y = -0.044\mathrm{x}T$ (-0.044 is the rate of change in the regression line produced using % CFU after desiccation in Figure 2B)
Long term differences in Time (days) $\delta T=T_f - T_5$
Long term differences in survival (CFU) $\delta Y= Y_f - Y_5$

<u>Estimation of storage time:</u>

If:

$$Y = -0.044\mathrm{x}T \leftrightarrow$$
$$\delta Y = -0.044\mathrm{x}\delta T \leftrightarrow$$
$$Y_f - Y_5 = -0.044\mathrm{x}(T_f - T_5) \leftrightarrow$$
$$Y_f - Y_5 = -0.044\mathrm{x}(T_f - 5) \leftrightarrow$$
$$(Y_f - Y_5)/-0.044) + 5 = T_f$$

And:

$$Y_f - Y_5 = \%\ CFU_{Tf} - \%\ CFU_{T5} \leftrightarrow$$
$$Y_f - Y_5 = (N_f/N_0)\mathrm{x}100 - (N_5/N_0)\mathrm{x}100$$

Then the following formula can be used to estimate the storage time:

$$T_f = N_f/(N_0/IR)/-0.044) + 5$$

Estimation of the VBNC fraction

Relative to the fraction of green cells:

$$\%VBNC_g = 100-((N_f/N_0)*100)/((N_g/N_t)*100))*100 \leftrightarrow$$

$$\%VBNC_g = 100-((N_f/N_0)/(N_g/N_t))*100$$

Relative to the total number of cells:

$$\%VBNC_t = 100-((N_f/N_0)*100)/((N_t/N_t)*100))*100 \leftrightarrow$$

$$\%VBNC_t = 100-(N_f/N_0)*100$$
