# Supplementary material for: Desiccation induces viable but Non-Culturable cells in Sinorhizobium meliloti 1021
Source: AMB Express. 2012 Jan 20;2:6. doi: 10.1186/2191-0855-2-6 (PMC3293009; doi:10.1186/2191-0855-2-6)

Additional file 2:

Figure S1: Example of bac/light direct counting results. Syto9 and Propidium iodine stained cells stored at 100% RH (A) and after drying and rewetting (B). After drying and rewetting ~50% of the cells stain red. These cells are in the process of dividing at the moment of drying (see white arrow). Direct microscopic observation does not indicate a change in cell morphology.

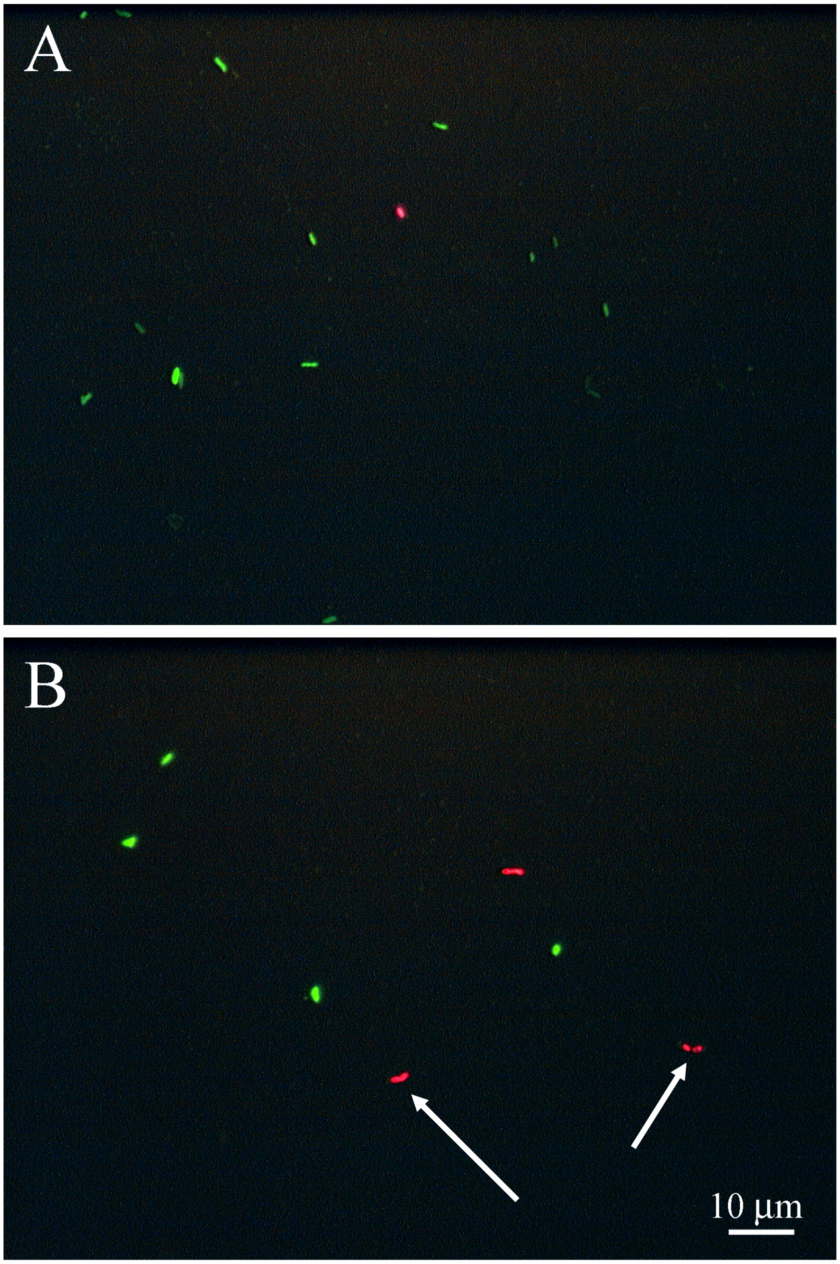

Supplement: Additional file 2 — Example of bac/light direct counting results. Photographic image of Syto9 and Propidium iodine stained cells stored at 100% RH (A) and after drying and rewetting (B). [file 2191-0855-2-6-S2.PDF]
